# Association between the triglyceride glucose index and the risk of acute kidney injury in critically ill patients with hypertension: analysis of the MIMIC-IV database

**DOI:** 10.3389/fendo.2024.1437709

**Published:** 2024-07-12

**Authors:** Wenbin Zhang, Zewen Yang

**Affiliations:** ^1^ Department of Endocrinology, Yiwu Traditional Chinese Medicine Hospital, Yiwu, Zhejiang, China; ^2^ Department of Cardiology, Yiwu Central Hospital, Yiwu, Zhejiang, China

**Keywords:** triglyceride-glucose index, insulin resistance, hypertension, acute kidney injury, MIMIC-IV database

## Abstract

**Background:**

The triglyceride glucose (TyG) index, a metric computed from the levels of fasting triglyceride (TG) and fasting plasma glucose (FPG), has emerged as a simple surrogate measure for insulin resistance (IR) in recent years. In multiple critical care scenarios, such as contrast-induced acute kidney injury (AKI) and cardiorenal syndrome, a high TyG index levels shows a notable correlation with AKI incidence. However, its predictive value for AKI in critically ill hypertensive patients remains uncertain.

**Methods:**

Participants were selected from the Medical Information Mart for Intensive Care IV (MIMIC-IV) database and divided into quartiles based on the TyG index. The primary focus of the study was to investigate the risk of acute kidney injury (AKI), with in-hospital mortality as a secondary endpoint, assessed among all study subjects as well as specifically among AKI patients. The use of renal replacement therapy (RRT), indicative of AKI progression, was also considered a secondary endpoint reflecting renal outcomes. To explore the correlation between the TyG index and AKI risk in critically ill hypertensive patients, the study employed a restricted cubic splines model and Cox proportional hazards (CPH) models. Additionally, Kaplan-Meier survival analysis was utilized to assess differences in primary and secondary outcomes across groups categorized by their TyG index. Analyses were conducted to ensure the consistency of the predictive capability of TyG index across various subgroups.

**Results:**

Our study included 4,418 participants, with 57% being male patients. AKI occurred in 56.1% of cases. Through the CPH analysis, we identified a significant association between the TyG index and AKI occurrence in critically ill hypertensive patients. With the help of a restricted cubic splines model, we observed a direct relationship between an elevated TyG index and an increased AKI. Subgroup examinations consistently proved the predictive value of the TyG index across categories. Furthermore, Kaplan-Meier survival analysis revealed notable differences in RRT among AKI patients.

**Conclusion:**

The findings underscore the importance of the TyG index as a reliable predictor for the occurrence of AKI and adverse renal outcomes among hypertensive patients in critical ill states. Nevertheless, validating causality mandates extensive prospective investigations.

## 1 Introduction

Despite the introduction of many pharmaceuticals and devices for hypertension in recent years, hypertension remains the leading preventable cause to cardiovascular mortality and disease burden worldwide (1–3). Given that kidney is widely acknowledged as a target organ of hypertension, acute kidney injury (AKI) has emerged as a common complication among hypertensive patients, particularly those in the intensive care unit (ICU) (4, 5). In view of the consistent correlation between AKI and heightened death in hypertensive patients, it is crucial to prioritize the identification of hypertensive patients at high risk of AKI in the ICU to improve their prognosis.

Previous research has predominantly examined specific clinical biomarkers linked to AKI in hypertension and other conditions, including neutrophil gelatinase-associated lipocalin (NGAL), kidney injury marker 1 (KIM-1), cystatin C (CysC), ST2, interleukin 18 (IL-18), and albuminuria (6–9). However, the array of well-established AKI biomarkers for critically ill hypertensive patients remains constrained. Hence, there is an imperative to explore more effective risk stratification approaches for these patients and promptly implement preventive interventions.

Insulin resistance (IR), which entails reduced sensitivity and responsiveness to insulin, plays a crucial role in the onset of hypertension and kidney dysfunction (10–12). The triglyceride glucose (TyG) index, a combined measure computed from fasting TG and FPG levels (13), has become a straightforward indicator of IR (14). It has been proved to be effective in predicting the progression of chronic kidney disease (CKD), coronary artery diseases, diabetes mellitus (DM), heart failure, hypertension, and overall mortality (15–20). Of particular note, recent studies have shown a direct association between an elevated TyG and the AKI occurrence as well as long-term adverse outcomes in critical conditions like contrast-induced AKI and cardiorenal syndrome (21–23).

Nevertheless, significant ambiguity remains regarding the relationship between the TyG index and the occurrence of AKI in critically ill hypertensive patients. Therefore, this study seeks to elucidate the predictive significance of the TyG index in relation to AKI.

## 2 Materials and methods

### 2.1 Data source and study population

In this study, a retrospective observation method was utilized, and the data was obtained from MIMIC-IV (https://mimic.mit.edu). This database included over 40,000 patients in the ICU at Beth Israel Deaconess Medical Center from 2008 to 2019 (24). The research followed the STROCSS reporting guidelines. Zewen Yang obtained both a Collaborative Institutional Training Initiative (CITI) license and necessary permissions to access and use the database to ensure the compliance with pertinent regulations.

This study focuses on a subset of the MIMIC-IV database, comprising 27,793 patients with hypertension. They are admitted to the ICU and aged 18 years and older in a non-consecutive way. For individuals who had multiple stays in the ICU, only the first admission during their initial hospital stay was taken into account. To ensure data reliability, individuals were excluded if there was insufficient information on TG and glucose, or if AKI data within 48 hours of ICU admission was missing.

**Figure 1:**
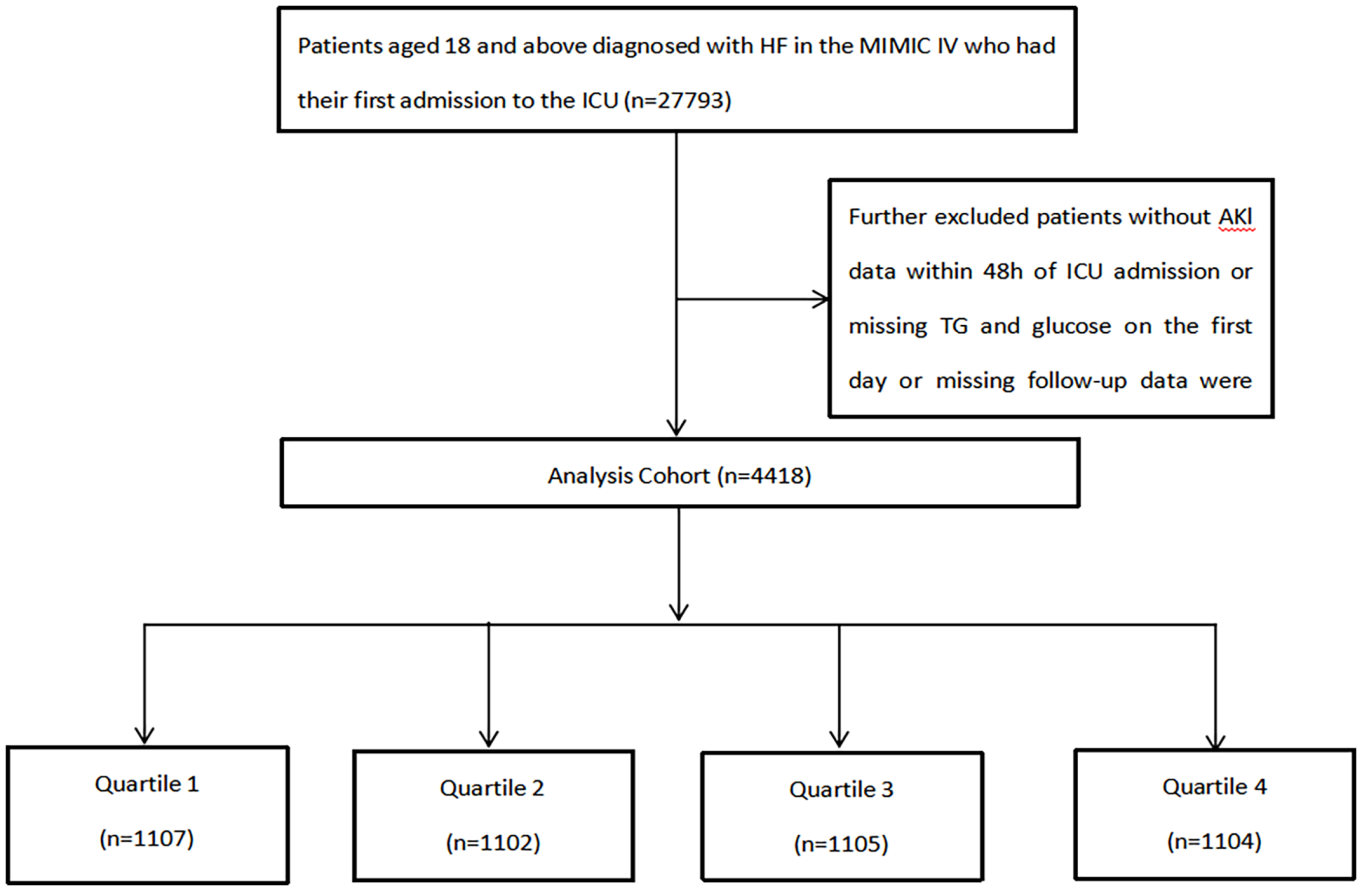
Flow chart of patient selection.

Ultimately, 4,418 eligible patients were categorized into four groups on their first day of ICU admission (**Figure 1**).

### 2.2 Data collection

Utilizing Structured Query Language (SQL) and PostgreSQL, we extracted baseline characteristics of the patients from MIMIC-IV. These characteristics included demographic details like age, gender, ethnicity, BMI; key health indicators such as SBP, DBP, and HR; medication specifics including ACEIs, ARBs, beta blockers, CCBs, statins, aspirin, as well as clopidogrel; measures of illness severity assessed by SIRS score, APSIII, SAPSII, and SOFA score. Laboratory findings including RBCs, WBCs, BUN, neutrophils, Hb, lymphocytes, platelets, Scr, TC, TG, HDL-C, NT-proBNP, LDL-C, PT, potassium, sodium, chloride, pH, PO_2_, PCO_2_, glucose, HbA1c, and TnT, were all documented within the initial 24 hours after the ICU admission. Additionally, details of surgical interventions such as PCI and CABG, as well as information on pre-existing medical conditions and in-hospital mortality outcomes, were extracted from the database.

Chronic obstructive pulmonary disease (COPD), percutaneous coronary intervention (PCI), acute myocardial infarction (AMI), DM, coronary artery bypass grafting (CABG), congestive heart failure (CHF), obstructive sleep apnea-hypopnea syndrome (OSAHS), and CKD were identified in adherence to the International Classification of Diseases, 10th Revision, and ICD-9 codes.

The follow-up period was calculated from the admission to the occurrence of the specified endpoints.

Multiple imputation was used to interpolate missing values. For variables with more than 25% missing data, dummy variables were created within the models to mitigate potential bias from inputting these missing values. The affected variables included BMI, SBP, DBP, HbA1c, HDL-C, LDL-C, PH, PCO_2_, PO_2_, lymphocytes, neutrophils, NT-proBNP, and TnT.

### 2.3 Endpoints of interest

The primary endpoint was AKI as per the guidelines of the kidney disease: Improving Global Outcomes Initiative. It was characterized by either an elevation in serum Scr levels to 1.5 times or more the baseline of the preceding week, or an increase of 0.3 mg/dl or more in Scr within 48 hours, or urine of less than 0.5 ml/kg/h for six or more hours.

The baseline Scr level was determined as the lowest Scr value recorded in the seven days prior to admission. If no Scr values were available before admission, the first Scr measurement upon admission was used as the baseline.

The secondary endpoint was in-hospital death for all entire subjects and the group affected by AKI. Additionally, the utilization of Renal Replacement Therapy (RRT), an indicator of the severity of AKI progression, was incorporated as a secondary endpoint to signify renal outcomes.

### 2.4 Statistical analysis

Continuous variables were presented through the mean accompanied by standard deviation (SD) or the median along with interquartile range (IQR). Group comparisons were conducted through the Mann-Whitney U test or the student t-test, according to the distribution of the data. Categorical variables were represented as frequencies and percentages and group results were compared through Fisher’s exact test or Pearson’s chi-square test. Kaplan-Meier survival analysis was employed to evaluate the occurrence of AKI, the use of RRT, and in-hospital mortality across groups, stratified by the TyG index and various states of glucose metabolism.

CPH models were used to determine the HR and 95% CI for the TyG index and its association with AKI across groups, with adjustments for many variables. Model 1 depicted an analysis without adjustments, whereas Model 2 accounted for gender, age, race, and BMI. Model 3 expanded on the Model 2 by including additional factors such as BUN, SCR, SOFA, SIRS, WBC, HR, DM, CKD, CHF, ACEI, ARB, PCI, CABG, Hb, SBP, and DBP. The analysis incorporated both continuous and categorical forms of the TyG index. HRs were calculated and presented with their 95% CIs. In all analyses, the group in the lowest quartile of the TyG index served as the reference.

Furthermore, a restricted cubic splines model was utilized to explore the dose-response relationship between the TyG index and the AKI occurrence. This analysis was adjusted for models described above.

The consistency of the prognostic significance of the TyG index was assessed through subgroup analyses. The subjects in our study were categorized based on age (<65 vs. ≥65 years), gender (female vs. male), BMI (underweight: <18.5, normal: 18.5–23.9, overweight: 23.9–29.9, obesity: ≥30 kg/m²), and the presence of ACEI, AMI, ARB, CHF, CKD, and DM. Likelihood ratio tests were performed to explore the correlation between the TyG index and variables employed for classification.

The statistical analyses were carried out through R version 4.2.2. A two-sided p-value of less than 0.05 was considered statistically significant.

## 3 Results

4,418 patients were investigated. The median age was 67 years [57, 78], and 2,501 patients (57%) were male. The median TyG index was 4.80 [4.58, 5.06]. The incidence rate of AKI was 57%.

### 3.1 Baseline characteristics

**Supplementary Table 1** displays the reference characteristics of the patients categorized in four cohorts according to the TyG index (Q1: 2.39-4.58; Q2: 4.58-4.80; Q3: 4.80-5.06; Q4: 5.06-7.28). The median of the TyG index were 4.45 [4.34, 4.52], 4.69 [4.64, 4.74], 4.92 [4.85, 4.98], and 5.29 [5.16, 5.51], respectively, in four groups. In the Q4 cohort, notable observations include a predominance of younger individuals, a larger proportion of male patients, a greater representation of Caucasians, an increased prevalence of obesity, and elevated scores indicating greater severity upon admission. Furthermore, this group exhibited heightened incidences of AMI, PCI, DM, COPD, OSAHS and RRT. They also demonstrated elevated levels of HR, BUN, glucose, platelets, potassium, Scr, TC, TG, RBC, WBC, as well as decreased levels of chlorine, Hb, PT and sodium.

What’s more, in the Q4 cohort, patients exhibited some specific clinical parameters: pH below 7.35, HDL cholesterol below 45 mg/dL, LDL cholesterol equal to or below 129 mg/dL, HbA1c above 6.4%, lymphocyte percentages below 18%, neutrophil percentages above 71%, PCO_2_ ranging between 35 and 45 mmHg, and PO_2_ levels above 105 mmHg. All are more prevalent. Moreover, there was a reduced utilization of aspirin in this cohort compared with that with lower TyG indices. The utilization of beta-blockers and statins also differed significantly among the groups (all with a level of P<0.05). Additionally, a heightened TyG index correlated with a progressively higher incidence of acute kidney injury (47% vs. 54% vs. 61% vs. 66%, P<0.001).

**Supplementary Table 2** presents a comparative analysis of baseline characteristics between patients with and without AKI. The RRT incidence rate was 6.6% in the AKI cohort. Those diagnosed with AKI include more male and Caucasian patients, as well as elevated HR values. There was also a higher incidence of AMI, CHF, CKD, COPD, DM, OSAHS, CABG, RRT but a lower PCI incidence in these patients. Greater use of betablocker and CCB, but lower use of ACEI, aspirin, clopidogrel and statin were found in the AKI group. As for laboratory indicators, AKI patients had elevated BUN, glucose, potassium, PT, Scr, TC, TG and WBC, but lower Hb, platelets and RBC (all with a significance level of P<0.05). Notably, the group exhibited a higher incidence of the following characteristics: HbA1c levels between 5.7% and 6.4%, HDL < 45 mg/dL, LDL ≤ 129 mg/dL, lymphocyte percentage < 18%, neutrophil percentage > 71%, and pH < 7.35 (all with a significance level of P < 0.05). Additionally, SOFA scores, SIRS scores, SAPS II, and APS III were significantly elevated in the AKI cohort (all with a significance level of P < 0.05). The AKI group also demonstrated a significantly higher TyG index compared to the other groups (4.84[4.63, 5.12] vs 4.73 [4.53, 4.98], P<0.001).

### 3.2 Primary endpoint

**Figure 2:**
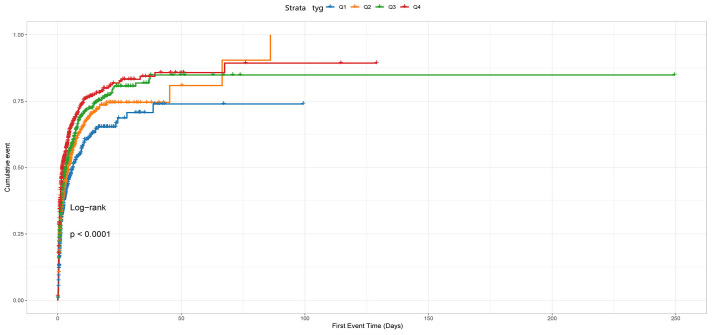
The cumulative event incidence curves for incidence of AKI. Notes: TyG index quartile Q1: 2.39–4.58; Q2: 4.58–4.80; Q3: 4.80–5.06; Q4: 5.06–7.28.

The cumulative incidence curve depicted in **Figure 2** demonstrates that patients with a TyG index ranging from 5.06 to 7.28 experienced the highest risk of AKI (Log-rank P<0.0001).

In this study, the Q1 group (TyG: 2.39-4.58) exhibited the lowest AKI incidence at 47% and was therefore selected as the reference group. CPH analysis indicated a significant association between AKI risk and the TyG index in both unadjusted models (HR 1.576, 95% CI 1.443-1.720, P<0.001) and adjusted models (HR 1.756, 95% CI 1.500-2.057, P<0.001) when the TyG index was deemed a continuous variable.

Furthermore, we found the Q4 group (TyG: 5.06~7.28) had the highest risk of AKI in both unadjusted models (Q1 vs. Q2: HR, 1.190 [95% CI 1.058–1.338] P=0.004; Q3: HR, 1.354 [95% CI 1.207–1.518] P<0.001; Q4: HR, 1.654 [95% CI 1.477–1.851] P<0.001) and adjusted models (Q1 vs. Q2: HR,1.313[95% CI 1.090, 1.583] P=0.004; Q3: HR, 1.454 [95% CI 1.203–1.758] P<0.001; Q4: HR, 1.944 [95% CI 1.609–2.348] P<0.001), with the TyG index being a nominal variable.

Additionally, we noted a substantial linear relationship between the TyG index and the AKI risk when conducting trend analyses in unadjusted and adjusted models considering various confounding factors (both with a P-value for trend < 0.001) as shown in **Table 1**.

**Table 1:** Cox proportional hazard ratios for AKI.

Categories	Model 1		P for trend	Model 2		P for trend	Model 3		P for trend
	HR (95% CI)	P*-*value		HR (95% CI)	P*-*value		HR (95% CI)	P*-*value	
AKI incidence									
Continuous variable per 1 unit	1.576 [95% CI 1.443, 1.720]	<0.001	<0.001	1.680 [95% CI 1.465, 1.926]	<0.001	<0.001	1.756 [95%CI 1.500, 2.057]	<0.001	<0.001
Quartile^1^									
Q1(N=1107)	Ref.			Ref.			Ref.		
Q2(N=1102)	1.190 [95% CI 1.058, 1.338]	0.004		1.314 [95% CI 1.091, 1.583]	0.004		1.313 [95% CI 1.090, 1.583]	0.004	
Q3(N=1105)	1.354 [95% CI 1.207, 1.518]	<0.001		1.458 [95% CI 1.210, 1.756]	<0.001		1.454 [95% CI 1.203, 1.758]	<0.001	
Q4(N=1104)	1.654 [95% CI 1.477, 1.851]	<0.001		1.913 [95% CI 1.594, 2.295]	<0.001		1.944 [95% CI 1.609, 2.348]	<0.001	

^1^TyG index quartile: Q1: 2.39–4.58; Q2: 4.58–4.80; Q3: 4.80–5.06; Q4: 5.06–7.28; Model 1 remained unaltered; Model 2 underwent adjustments in terms of age, gender, BMI, and race; Model 3 underwent adjustments that included the variables from Model 2 and additionally accounted for BUN, SCR, SOFA, SIRS, WBC, HR, DM, CKD, CHF, ACEI, ARB, PCI, CABG, Hb, SBP, and DBP.

**Figure 3:**
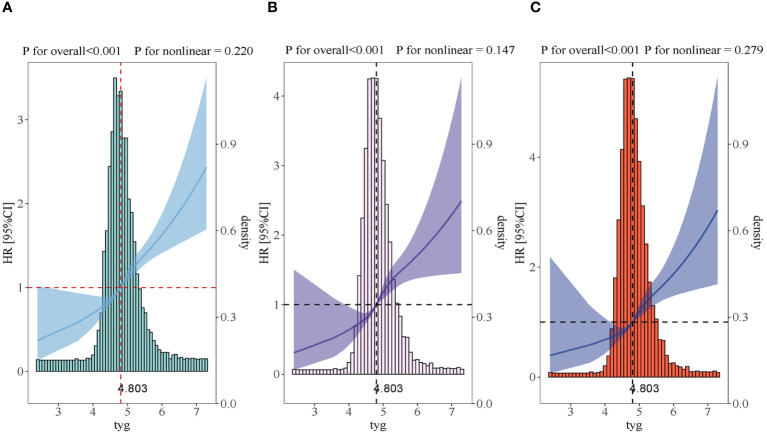
Restricted cubic spline curves for the TyG index hazard ratio. **(A)** Model 1 was not adjusted. **(B)** Model 2 was adjusted in terms of age, gender, BMI and race. **(C)** Model 3 expanded on model 2 and further adjusted for BUN, SCR, SOFA, SIRS, WBC, HR, DM, CKD, CHF, ACEI, ARB, PCI, CABG, Hb, SBP and DBP.

A dose-response relationship was observed between the TyG index and the AKI risk in all restricted cubic splines regression models (P for overall<0.001, P for non-linearity=0.220 and P for overall<0.001, P for non-linearity=0.279) and the AKI risk significantly increased when the TyG index was above 4.803 (**Figure 3**).

Extensive examination revealed the correlation between the TyG index and the main outcome across subcategories determined by age, gender, ACEI, AMI, ARB, BMI, CHF, CKD, DM, and HbA1c.

The TyG index exhibited a significant correlation with an elevated AKI in cohorts delineated by female [HR (95% CI) 1.75 (1.52–2.03)], male [HR (95% CI) 1.47 (1.32–1.65)], age>65 years [HR (95% CI) 1.83(1.58–2.11)], age<=65 years [HR (95% CI) 1.48 (1.32–1.67)], HbA1c<5.7 (HR [95% CI] = 1.86 [1.38, 2.51]), HbA1c>6.4 (HR [95% CI]= 1.26 [1.01, 1.55]), HbA1c 5.7–6.4 (HR [95% CI] =1.65 [1.31, 2.09]), normal group (HR [95% CI] =1.72 [1.17–2.01]), obesity group (HR [95% CI]=1.67 [1.35–2.06]), BMI overweight group (HR [95% CI]=1.67 [1.35–2.06]) and BMI underweight group (HR [95% CI]=6.16[1.91–19.87]), presence of DM (HR[95% CI]=1.57 [1.36–1.81]), absence of DM (HR [95%CI]=1.81 [1.61–2.04]), absence of CKD (HR [95% CI]=1.58 [1.45,1.73]), presence of AMI (HR [95% CI]=1.39 [1.10, 1.75]), and absence of AMI (HR[95% CI]=1.60 [1.46, 1.76]), presence of CHF (HR [95% CI]=1.41 [1.11,1.79]), and absence of CHF (HR [95% CI]=1.61 [1.46, 1.77]), medication history with ACEI(HR [95% CI] =1.36 [1.18, 1.58]), without ACEI (HR [95% CI]=1.72[1.55,1.92]),without ARB (HR [95% CI]=1.61 [1.47, 1.77]) (P<0.05).

**Figure 4:**
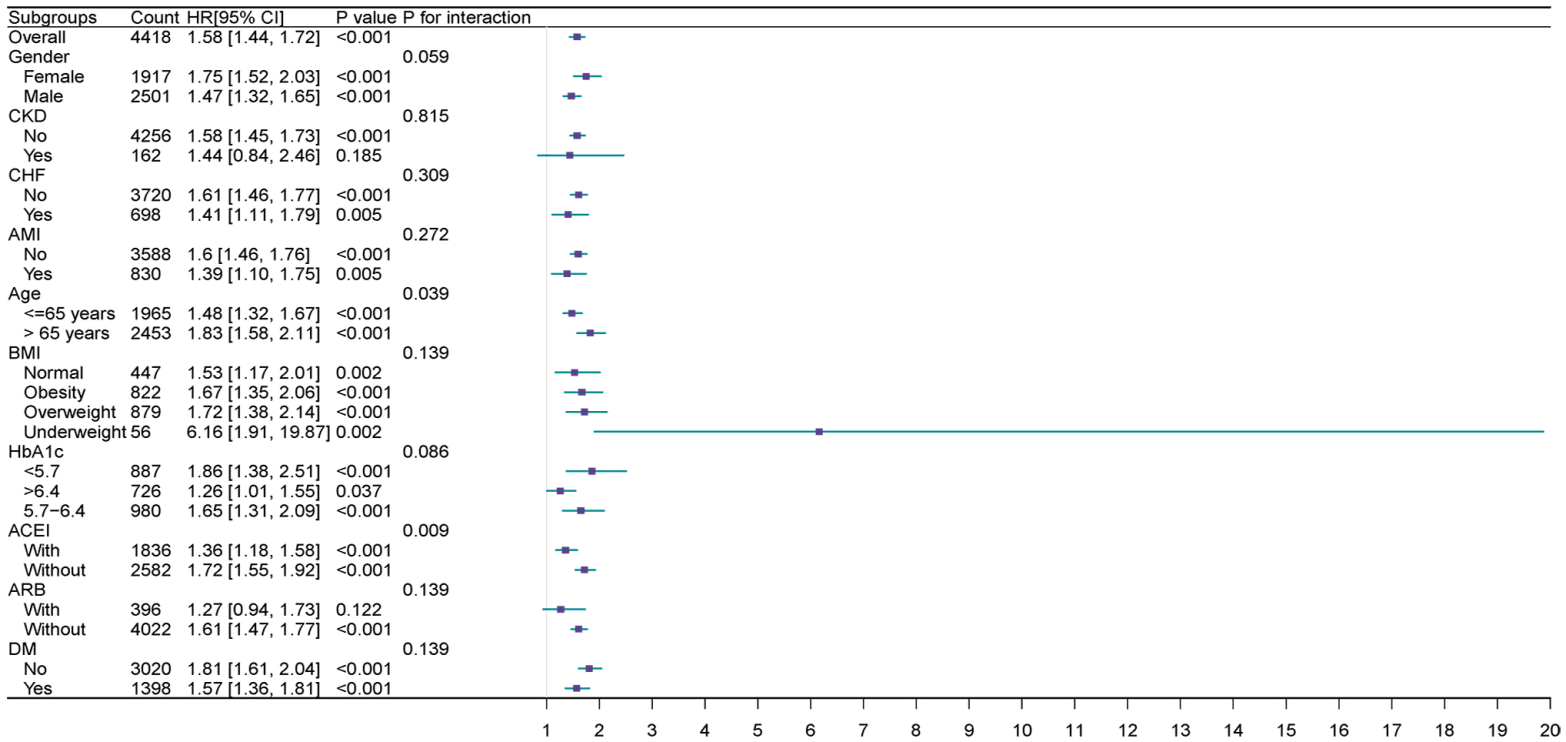
Forest plots of hazard ratios for the primary endpoint in different subgroups.

Furthermore, a significant correlation was noted between the TyG index and medication history, whether with or without ACE inhibitors (interaction P=0.009). This association was also observed in subgroups aged over 65 and those aged 65 or younger (interaction P=0.039) (**Figure 4**).

### 3.3 Secondary endpoints

**Figure 5:**
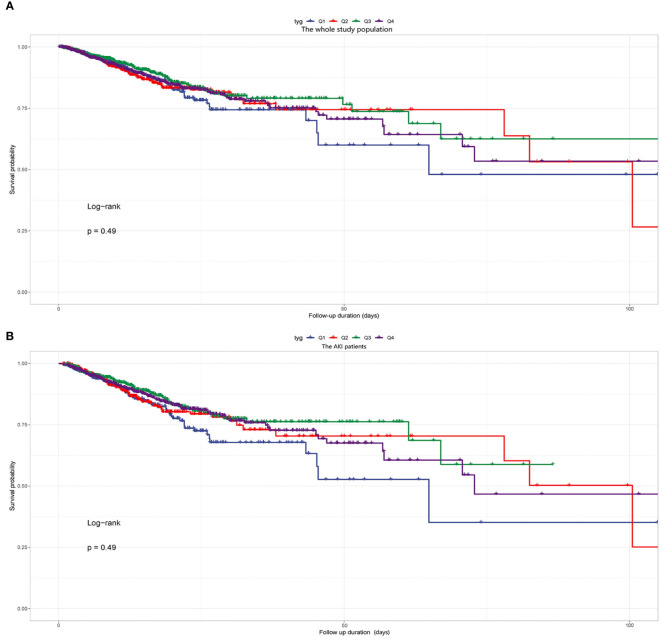
**(A)** Kaplan-Meier survival analysis curve for the in-hospital mortality of the whole study population. **(B)** Kaplan-Meier survival analysis curve for the in-hospital mortality of the AKI patients. Notes: TyG index quartile Q1: 2.39–4.58; Q2: 4.58–4.80; Q3: 4.80–5.06; Q4: 5.06–7.28.

**Figure 6:**
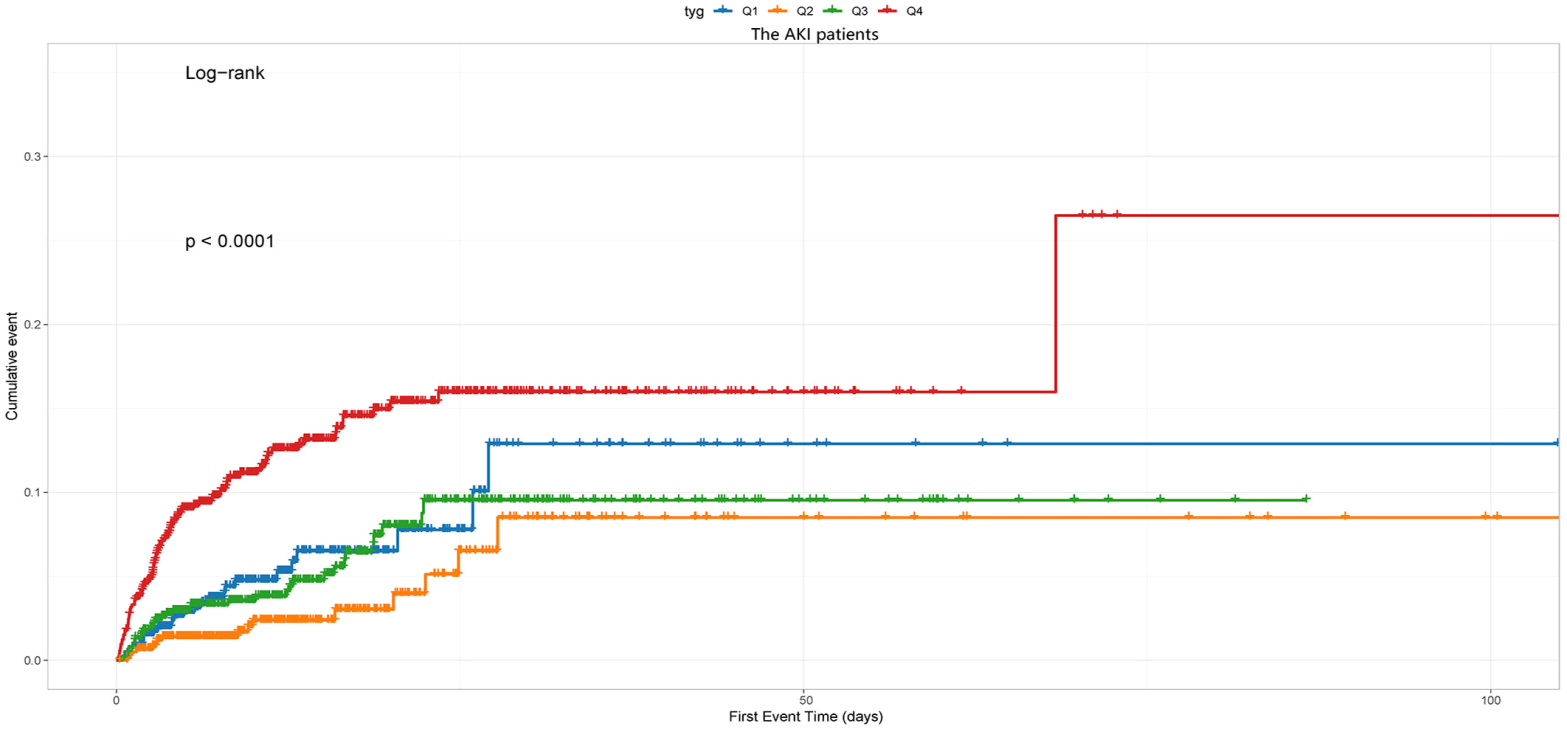
The cumulative event incidence curves for the use of RRT of the AKI patients. Notes: TyG index quartile Q1: 2.39–4.58; Q2: 4.58–4.80; Q3: 4.80–5.06; Q4: 5.06–7.28.

Kaplan-Meier survival analyses were conducted to assess how the TyG index influences the secondary endpoints within both the entire group and that diagnosed with the AKI. Our examination unveiled no statistically significant disparities in in-hospital death between the entire cohort (P=0.49, **Figure 5A**) and the AKI cohort (P=0.49, **Figure 5B**). Our investigation revealed a significantly higher RRT incidence among patients in the fourth quartile (P<0.001, **Figure 6**).

## 4 Discussion

This study represents the first comprehensive analysis investigating the correlation between the TyG index and the risk of AKI in critically ill hypertensive patients, employing various methodologies. Analyzing clinical data from 4,418 hypertensive individuals, we identified a robust association between the TyG index and AKI risk. This association persisted even after accounting for potential confounding factors. Furthermore, the TyG index demonstrated significant predictive value for AKI progression to RRT in critically ill hypertensive patients. Importantly, this study introduces a straightforward approach for assessing IR, which could improve the stratification of AKI risk in this patient cohort.

### 4.1 TyG index, hypertension, and kidney disease risk

The TyG index features high sensitivity, specificity, convenience, and low cost, and is widely regarded as a reliable index in the evaluation of IR (25, 26). The link of the TyG index and hypertension has been widely and comprehensively discussed. Plenty of cross-sectional studies have consistently shown an association between an elevated TyG index and increased incidence of hypertension across various demographic groups. For example, Zheng et al. conducted a longitudinal study involving 4,686 subjects over a period of 9 years, demonstrating that a higher TyG index was associated with an elevated risk of developing hypertension within the general Chinese population (27). Simental-Mendia et al. also found in a cross-sectional study that a heightened TyG index has significant correlation with hypertension in children aged 6–9 and adolescents aged 10–15 (13). Moreover, several investigations have suggested that the TyG index may predict adverse outcomes in individuals with hypertension. A recent prospective study using NHANES data underscored a non-linear association between the TyG index and both cardiovascular and all-cause mortality among hypertensive patients (28, 29). Similarly, Pan et al. uncovered an L-shaped connection between the TyG index and the probability of all-cause mortality in the middle-aged and elderly patients diagnosed with hypertension (28, 29).

In the field of kidney diseases, Setor and colleagues identified a significant correlation between an elevated TyG index and increased susceptibility to renal dysfunction in a cohort comprising 2,362 men aged 42–61 with initially normal kidney function. This association was observed throughout an average follow-up period of 17.5 years. Additionally, another study involving 1,936 patients diagnosed with Type 2 diabetes mellitus and chronic kidney disease demonstrated a notable positive correlation, highlighting that individuals with a higher TyG index faced the greatest risk of progressing to end-stage renal disease (18). Furthermore, our group recently proved that the TyG index could forecast AKI and adverse renal outcomes in critically ill patients with HF. Similar findings were also reported in a study by Jin et al. involving a general ICU population (22, 30).

The foregoing evidence proved that the TyG index is reliable and accurate in identifying insulin resistance, thereby aiding in stratifying the AKI risk in critically ill hypertensive patients.

### 4.2 The mechanisms behind the correlation between IR, the TyG index, and AKI in hypertensive patients with severe illness

Our study unveils the significant independent predictive capability of the TyG index in forecasting AKI among patients with hypertension in the ICU. This is particularly notable given the scarcity of relative clinical data in this domain. The mechanisms through which IR triggers pathological interactions between hypertension and AKI likely involve several factors. Firstly, individuals with elevated TyG index levels may exhibit heightened susceptibility to lifestyle-related risk factors, underlying diseases, and diminished healthcare awareness. This association arises from the TyG index being a metric calculated from fasting levels of triglycerides and glucose. In our investigation, subjects with elevated TyG index values demonstrated a propensity towards overweight status and exhibited higher prevalence rates of conditions such as DM, hyperlipidemia, AMI, COPD and OSAHS. Secondly, the TyG index serves as a valuable biomarker for IR and its associated hyperinsulinemia. These conditions correlate with chronic inflammation, oxidative stress, mitochondrial dysfunction, and vascular wall injury. Research has shown that IR and hyperinsulinemia can lead to reduced nitric oxide (NO) production and increased oxidative destruction of NO, ultimately causing endothelial dysfunction and hypertension. Furthermore, IR and hyperinsulinemia associated with oxidative stress can lead to injury of glomerular endothelial cells, proliferation of mesangial cells, and thickening of basement membranes. Additionally, the insulin receptor on renal tubular cells and podocytes plays a crucial role in insulin signaling, influencing renal hemodynamics, podocyte viability, and tubular function. Defective insulin receptor signaling due to IR and hyperinsulinemia can lead to a pathological condition resembling diabetic nephropathy, even in the absence of elevated blood glucose levels. These physiological mechanisms collectively impact the development of hypertension and the progressive deterioration of kidney (31–37). Thirdly, IR and its associated hyperinsulinemia are closely associated with inappropriate activation of sympathetic nervous system and RAAS, which leads to higher angiotensin II levels (38, 39). Angiotensin II, in turn, heightens systemic pressure, glomerular hydrostatic pressure, and urinary albumin excretion, thereby causing the early glomerular hyperfiltration and subsequent glomerular damage during initial renal dysfunction (40–43). Our study indirectly corroborates this mechanism by demonstrating a significant AKI risk decrease with the use of ACEIs in subgroup analyses. Moreover, overstimulation of the sympathetic nervous system can increase cardiac workload and exacerbate vascular and renal dysfunction (44, 45). Fourthly, IR and its associated hyperinsulinemia are linked to increased reabsorption of sodium in the kidneys and higher levels of antidiuretic hormones, or relative insufficiency in natriuretic hormones, thereby exacerbating hypertension through the retention of water and sodium, ultimately leading to renal impairment (12, 46). On the other hand, IR and its associated hyperinsulinemia induce medial and intimal thickening in afferent arterioles. This thickening impairs the arterioles’ ability to contract and dilate effectively, resulting in inadequate control of glomerular pressure. Persistent elevation of glomerular pressure aggravates proteinuria and, ultimately, leads to reduced glomerular filtration rate due to mesangial denaturation or glomerular necrosis. These changes can accelerate the progression of renal damage (47, 48).. Additionally, reports indicate that IR synergistically interacts with elevated blood pressure, perpetuating a vicious cycle that exacerbates vascular and renal injuries. Consequently, this worsens hypertension and results in additional damage to the kidneys and cardiovascular system (49, 50).

### 4.3 Study limitations

This study has several limitations that merit consideration. Firstly, it is essential to note that the study was retrospective and observational, which precludes establishing definitive causal relationships. Secondly, despite employing multivariate adjustment and subgroup analyses, the study utilized a limited sample size, introducing a potential for data bias due to unaccounted confounding factors. Thirdly, certain factors like hypertension severity, chronic kidney disease, AKI progression to AKD, CKD, ESRD, and participants’ socioeconomic status weren’t taken into consideration. This limitation hinders a more nuanced examination of the connection between the TyG index and death across different hypertension grades. Fourthly, our investigation focused exclusively on assessing the prognostic value of the TyG index for AKI in critically ill hypertensive patients, without considering longitudinal changes in the TyG index. Lastly, further cohort studies are warranted to validate our findings.

## 5 Conclusions

Our research reveals a notable and independent correlation between heightened TyG index level and increased AKI risk in hypertensive patients in the ICU. Particularly noteworthy is the substantial predictive efficacy of NLR in anticipating AKI patients necessitating RRT. The TyG index could be an easy way to spot hypertensive patients with a higher AKI risk, facilitating targeted therapeutic interventions. Nevertheless, further research is imperative to corroborate the findings and understand the reasons behind them.

## Data availability statement

The original contributions presented in the study are included in the article/**Supplementary Material**. Further inquiries can be directed to the corresponding author.

## Author contributions

WZ: Writing – review & editing, Writing – original draft, Supervision, Methodology, Investigation, Formal analysis, Conceptualization. ZY: Writing – original draft, Resources, Methodology, Investigation, Formal analysis.

## References

[1] Roth GA Mensah GA Johnson CO Addolorato G Ammirati E Baddour LM. Global burden of cardiovascular diseases and risk factors, 1990-2019: update from the gbd 2019 study. J Am Coll Cardiol. (2020) 76:2982–3021. doi: 10.1016/j.jacc.2020.11.010 33309175 PMC7755038

[2] Lewington S Lacey B Clarke R Guo Y Kong XL Yang L. The burden of hypertension and associated risk for cardiovascular mortality in China. JAMA Intern Med. (2016) 176:524–32. doi: 10.1001/jamainternmed.2016.0190 26975032

[3] Li Y Yang L Wang L Zhang M Huang Z Deng Q. Burden of hypertension in China: A nationally representative survey of 174,621 adults. Int J Cardiol. (2017) 227:516–23. doi: 10.1016/j.ijcard.2016.10.110 27856040

[4] Zarbock A Weiss R Albert F Rutledge K Kellum JA Bellomo R. Epidemiology of surgery associated acute kidney injury (Epis-aki): A prospective international observational multi-center clinical study. Intensive Care Med. (2023) 49:1441–55. doi: 10.1007/s00134-023-07169-7 PMC10709241 37505258

[5] Sovik S Isachsen MS Nordhuus KM Tveiten CK Eken T Sunde K. Acute kidney injury in trauma patients admitted to the icu: A systematic review and meta-analysis. Intensive Care Med. (2019) 45:407–19. doi: 10.1007/s00134-019-05535-y 30725141

[6] Rubatto Birri PN Giannoni R Furche M Nahra M Arce Gallardo M Segui G. Epidemiology, patterns of care and prognosis of acute kidney injury in critically ill patients: A multicenter study in Argentina (the epira study). J Crit Care. (2023) 78:154382. doi: 10.1016/j.jcrc.2023.154382 37516091

[7] Griffin BR Liu KD Teixeira JP. Critical care nephrology: core curriculum 2020. Am J Kidney Dis. (2020) 75:435–52. doi: 10.1053/j.ajkd.2019.10.010 PMC7333544 31982214

[8] Malhotra R Siew ED. Biomarkers for the early detection and prognosis of acute kidney injury. Clin J Am Soc Nephrol. (2017) 12:149–73. doi: 10.2215/CJN.01300216 PMC5220647 27827308

[9] Koyner JL Vaidya VS Bennett MR Ma Q Worcester E Akhter SA. Urinary biomarkers in the clinical prognosis and early detection of acute kidney injury. Clin J Am Soc Nephrol. (2010) 5:2154–65. doi: 10.2215/CJN.00740110 PMC2994075 20798258

[10] Demir S Nawroth PP Herzig S Ekim Üstünel B. Emerging targets in type 2 diabetes and diabetic complications. Adv Sci (Weinh). (2021) 8:e2100275. doi: 10.1002/advs.202100275 34319011 PMC8456215

[11] Jia G Sowers JR. Hypertension in diabetes: an update of basic mechanisms and clinical disease. Hypertension. (2021) 78:1197–205. doi: 10.1161/HYPERTENSIONAHA.121.17981 PMC8516748 34601960

[12] Hall JE Mouton AJ da Silva AA Omoto ACM Wang Z Li X. Obesity, kidney dysfunction, and inflammation: interactions in hypertension. Cardiovasc Res. (2021) 117:1859–76. doi: 10.1093/cvr/cvaa336 PMC8262632 33258945

[13] Simental-Mendia LE Hernandez-Ronquillo G Gamboa-Gomez CI Gomez-Diaz R Rodriguez-Moran M Guerrero-Romero F. The triglycerides and glucose index is associated with elevated blood pressure in apparently healthy children and adolescents. Eur J Pediatr. (2019) 178:1069–74. doi: 10.1007/s00431-019-03392-x 31081518

[14] Guerrero-Romero F Simental-Mendía LE González-Ortiz M Martínez-Abundis E Ramos-Zavala MG Hernández-González SO. The product of triglycerides and glucose, a simple measure of insulin sensitivity. Comparison with the euglycemic-hyperinsulinemic clamp. J Clin Endocrinol Metab. (2010) 95:3347–51. doi: 10.1210/jc.2010-0288 20484475

[15] Kim MK Ahn CW Kang S Nam JS Kim KR Park JS. Relationship between the triglyceride glucose index and coronary artery calcification in Korean adults. Cardiovasc Diabetol. (2017) 16:108. doi: 10.1186/s12933-017-0589-4 28830471 PMC5568209

[16] Gao Q Lin Y Xu R Luo F Chen R Li P. Positive association of triglyceride-glucose index with new-onset hypertension among adults: A national cohort study in China. Cardiovasc Diabetol. (2023) 22:58. doi: 10.1186/s12933-023-01795-7 36927705 PMC10022268

[17] Huang R Wang Z Chen J Bao X Xu N Guo S. Prognostic value of triglyceride glucose (Tyg) index in patients with acute decompensated heart failure. Cardiovasc Diabetol. (2022) 21:88. doi: 10.1186/s12933-022-01507-7 35641978 PMC9158138

[18] Kunutsor SK Seidu S Kurl S Laukkanen JA. Baseline and usual triglyceride-glucose index and the risk of chronic kidney disease: A prospective cohort study. Geroscience. (2024) 46:3035–46. doi: 10.1007/s11357-023-01044-5 PMC11009217 38180700

[19] Liao Y Zhang R Shi S Zhao Y He Y Liao L. Triglyceride-glucose index linked to all-cause mortality in critically ill patients: A cohort of 3026 patients. Cardiovasc Diabetol. (2022) 21:128. doi: 10.1186/s12933-022-01563-z 35804386 PMC9270811

[20] Lee SH Park SY Choi CS. Insulin resistance: from mechanisms to therapeutic strategies. Diabetes Metab J. (2022) 46:15–37. doi: 10.4093/dmj.2021.0280 34965646 PMC8831809

[21] Qin Y Tang H Yan G Wang D Qiao Y Luo E. A high triglyceride-glucose index is associated with contrast-induced acute kidney injury in Chinese patients with type 2 diabetes mellitus. Front Endocrinol (Lausanne). (2020) 11:522883. doi: 10.3389/fendo.2020.522883 33551987 PMC7862330

[22] Yang Z Gong H Kan F Ji N. Association between the triglyceride glucose (Tyg) index and the risk of acute kidney injury in critically ill patients with heart failure: analysis of the mimic-iv database. Cardiovasc Diabetol. (2023) 22:232. doi: 10.1186/s12933-023-01971-9 37653418 PMC10472684

[23] Lv L Xiong J Huang Y He T Zhao J. Association between the triglyceride glucose index and all-cause mortality in critically ill patients with acute kidney injury. Kidney Dis (Basel). (2024) 10:69–78. doi: 10.1159/000535891 38322625 PMC10843181

[24] Johnson AE Pollard TJ Shen L Lehman LW Feng M Ghassemi M. Mimic-iii, a freely accessible critical care database. Sci Data. (2016) 3:160035. doi: 10.1038/sdata.2016.35 27219127 PMC4878278

[25] Unger G Benozzi SF Perruzza F Pennacchiotti GL. Triglycerides and glucose index: A useful indicator of insulin resistance. Endocrinol Nutr. (2014) 61:533–40. doi: 10.1016/j.endonu.2014.06.009 25174769

[26] Vasques AC Novaes FS de Oliveira Mda S Souza JR Yamanaka A Pareja JC. Tyg index performs better than homa in a Brazilian population: A hyperglycemic clamp validated study. Diabetes Res Clin Pract. (2011) 93:e98–e100. doi: 10.1016/j.diabres.2011.05.030 21665314

[27] Zheng R Mao Y. Triglyceride and glucose (Tyg) index as a predictor of incident hypertension: A 9-year longitudinal population-based study. Lipids Health Dis. (2017) 16:175. doi: 10.1186/s12944-017-0562-y 28903774 PMC5598027

[28] Zhou D Liu XC Kenneth L Huang YQ Feng YQ. A non-linear association of triglyceride glycemic index with cardiovascular and all-cause mortality among patients with hypertension. Front Cardiovasc Med. (2021) 8:778038. doi: 10.3389/fcvm.2021.778038 35155598 PMC8828937

[29] Pang J Qian L Che X Lv P Xu Q. Tyg index is a predictor of all-cause mortality during the long-term follow-up in middle-aged and elderly with hypertension. Clin Exp Hypertens. (2023) 45:2272581. doi: 10.1080/10641963.2023.2272581 37902269

[30] Jin Z Zhang K. Association between triglyceride-glucose index and aki in icu patients based on mimiciv database: A cross-sectional study. Ren Fail. (2023) 45:2238830. doi: 10.1080/0886022X.2023.2238830 37563796 PMC10424620

[31] Tang SCW Yiu WH. Innate immunity in diabetic kidney disease. Nat Rev Nephrol. (2020) 16:206–22. doi: 10.1038/s41581-019-0234-4 31942046

[32] Kaludercic N Di Lisa F. Mitochondrial ros formation in the pathogenesis of diabetic cardiomyopathy. Front Cardiovasc Med. (2020) 7:12. doi: 10.3389/fcvm.2020.00012 32133373 PMC7040199

[33] Wang M Li Y Li S Lv J. Endothelial dysfunction and diabetic cardiomyopathy. Front Endocrinol (Lausanne). (2022) 13:851941. doi: 10.3389/fendo.2022.851941 35464057 PMC9021409

[34] Yamamoto Y Doi T Kato I Shinohara H Sakurai S Yonekura H. Receptor for advanced glycation end products is a promising target of diabetic nephropathy. Ann N Y Acad Sci. (2005) 1043:562–6. doi: 10.1196/annals.1333.064 16037279

[35] Ohishi M. Hypertension with diabetes mellitus: physiology and pathology. Hypertens Res. (2018) 41:389–93. doi: 10.1038/s41440-018-0034-4 29556093

[36] Sahakyan G Vejux A Sahakyan N. The role of oxidative stress-mediated inflammation in the development of T2dm-induced diabetic nephropathy: possible preventive action of tannins and other oligomeric polyphenols. Molecules. (2022) 27:9035. doi: 10.3390/molecules27249035 36558167 PMC9786776

[37] Forbes JM Cooper ME. Mechanisms of diabetic complications. Physiol Rev. (2013) 93:137–88. doi: 10.1152/physrev.00045.2011 23303908

[38] Zhou MS Schulman IH Zeng Q. Link between the renin-angiotensin system and insulin resistance: implications for cardiovascular disease. Vasc Med. (2012) 17:330–41. doi: 10.1177/1358863X12450094 22814999

[39] Wolf G Ziyadeh FN. The role of angiotensin ii in diabetic nephropathy: emphasis on nonhemodynamic mechanisms. Am J Kidney Dis. (1997) 29:153–63. doi: 10.1016/S0272-6386(97)90023-8 9002545

[40] Mogensen CE. Glomerular filtration rate and renal plasma flow in short-term and long-term juvenile diabetes mellitus. Scand J Clin Lab Invest. (1971) 28:91–100. doi: 10.3109/00365517109090667 5093523

[41] Patel S Rauf A Khan H Abu-Izneid T. Renin-angiotensin-aldosterone (Raas): the ubiquitous system for homeostasis and pathologies. BioMed Pharmacother. (2017) 94:317–25. doi: 10.1016/j.biopha.2017.07.091 28772209

[42] Parving HH Andersen S Jacobsen P Christensen PK Rossing K Hovind P. Angiotensin receptor blockers in diabetic nephropathy: renal and cardiovascular end points. Semin Nephrol. (2004) 24:147–57. doi: 10.1016/j.semnephrol.2003.11.003 15017527

[43] Te Riet L van Esch JH Roks AJ van den Meiracker AH Danser AH. Hypertension: renin-angiotensin-aldosterone system alterations. Circ Res. (2015) 116:960–75. doi: 10.1161/CIRCRESAHA.116.303587 25767283

[44] Lambert GW Straznicky NE Lambert EA Dixon JB Schlaich MP. Sympathetic nervous activation in obesity and the metabolic syndrome–causes, consequences and therapeutic implications. Pharmacol Ther. (2010) 126:159–72. doi: 10.1016/j.pharmthera.2010.02.002 20171982

[45] Thorp AA Schlaich MP. Relevance of sympathetic nervous system activation in obesity and metabolic syndrome. J Diabetes Res. (2015) 2015:341583. doi: 10.1155/2015/341583 26064978 PMC4430650

[46] Gluba A Mikhailidis DP Lip GY Hannam S Rysz J Banach M. Metabolic syndrome and renal disease. Int J Cardiol. (2013) 164:141–50. doi: 10.1016/j.ijcard.2012.01.013 22305775

[47] Tonneijck L Muskiet MH Smits MM van Bommel EJ Heerspink HJ van Raalte DH. Glomerular hyperfiltration in diabetes: mechanisms, clinical significance, and treatment. J Am Soc Nephrol. (2017) 28:1023–39. doi: 10.1681/ASN.2016060666 PMC5373460 28143897

[48] Anderson S Meyer TW Rennke HG Brenner BM. Control of glomerular hypertension limits glomerular injury in rats with reduced renal mass. J Clin Invest. (1985) 76:612–9. doi: 10.1172/JCI112013 PMC423867 2993362

[49] Artunc F Schleicher E Weigert C Fritsche A Stefan N Haring HU. The impact of insulin resistance on the kidney and vasculature. Nat Rev Nephrol. (2016) 12:721–37. doi: 10.1038/nrneph.2016.145 27748389

[50] Hall JE do Carmo JM da Silva AA Wang Z Hall ME. Obesity, kidney dysfunction and hypertension: mechanistic links. Nat Rev Nephrol. (2019) 15:367–85. doi: 10.1038/s41581-019-0145-4 PMC7278043 31015582

