# Supplementary material for: Association between the triglyceride glucose index and the risk of acute kidney injury in critically ill patients with hypertension: analysis of the MIMIC-IV database
Source: Front Endocrinol (Lausanne). 2024 Jul 12;15:1437709. doi: 10.3389/fendo.2024.1437709 (PMC11272463; doi:10.3389/fendo.2024.1437709)
Supplement: Supplementary file 1 [file Table_1.docx]

Supplementary Material

# Supplementary Tables

## Supplementary Table 1. Baseline characteristics categorized by quartiles of the TyG index^1^.

| **Variables** | **Overall** | **Q1** | **Q2** | **Q3** | **Q4** | **P-value^2^** |
| --- | --- | --- | --- | --- | --- | --- |
| Patient number | 4,418 | 1107 | 1102 | 1105 | 1104 |  |
| Male (n%) | 2,501 (57%) | 591 (53%) | 614 (56%) | 625 (57%) | 671 (61%) | 0.005** |
| Age, years, (median [IQR]) | 67 [57, 78] | 72 [61, 82] | 71 [60, 80] | 67 [57, 77] | 61 [52, 70] | <0.001*** |
| Race, n (%) |  |  |  |  |  | <0.001*** |
| Asian | 123 (2.8%) | 30 (2.7%) | 31 (2.8%) | 27 (2.4%) | 35 (3.2%) |  |
| Black | 407 (9.2%) | 133 (12%) | 109 (9.9%) | 83 (7.5%) | 82 (7.4%) |  |
| White | 2,799 (63%) | 712 (64%) | 711 (65%) | 703 (64%) | 673 (61%) |  |
| Other | 1,089 (25%) | 232 (21%) | 251 (23%) | 292 (26%) | 314 (28%) |  |
| BMI, kg/m2, n (%) |  |  |  |  |  | <0.001*** |
| Normal (18.5~23.9) | 447 (20%) | 150 (28%) | 133 (24%) | 99 (19%) | 65 (11%) |  |
| Obesity  (≥30) | 822 (37%) | 134 (25%) | 197 (35%) | 198 (38%) | 293 (51%) |  |
| Overweight (23.9~29.9) | 879 (40%) | 240 (44%) | 214 (38%) | 217 (41%) | 208 (36%) |  |
| Underweight (<18.5) | 56 (2.5%) | 21 (3.9%) | 19 (3.4%) | 11 (2.1%) | 5 (0.9%) |  |
| Missing | 2,214 | 562 | 539 | 580 | 533 |  |
| Disease severity score |  |  |  |  |  |  |
| APSIII (median [IQR]) | 36 [28, 47] | 34 [27, 43] | 35 [27, 43] | 36 [28, 48] | 39 [29, 55] | <0.001*** |
| SAPSII (median [IQR]) | 32 [26, 39] | 31 [27, 37] | 32 [26, 38] | 32 [26, 40] | 33 [25, 42] | 0.033* |
| SOFA (median [IQR]) | 3.0 [1.9, 5.0] | 3.0 [1.8, 4.3] | 3.0 [1.8, 4.9] | 3.0 [2.0, 5.0] | 4.0 [2.0, 7.0] | <0.001*** |
| SIRS (median [IQR]) | 2.30 [2.00, 3.00] | 2.00 [2.00, 3.00] | 2.11 [2.00, 3.00] | 2.53 [2.00, 3.00] | 3.00 [2.00, 3.00] | <0.001*** |
| Vital Signs |  |  |  |  |  |  |
| SBP, mmHg, n (%) |  |  |  |  |  | 0.062 |
| <=140 | 1,821 (74%) | 442 (72%) | 478 (76%) | 461 (76%) | 440 (71%) |  |
| >140 | 640 (26%) | 169 (28%) | 149 (24%) | 142 (24%) | 180 (29%) |  |
| Missing | 1,957 | 496 | 475 | 502 | 484 |  |
| DBP, mmhg, n (%) |  |  |  |  |  | 0.7 |
| <=90 | 2,227 (90%) | 556 (91%) | 571 (91%) | 546 (91%) | 554 (89%) |  |
| >90 | 234 (9.5%) | 55 (9.0%) | 56 (8.9%) | 57 (9.5%) | 66 (11%) |  |
| Missing | 1,957 | 496 | 475 | 502 | 484 |  |
| HR (median [IQR]) | 84 [76, 95] | 82 [74, 92] | 83 [75, 92] | 85 [76, 96] | 87 [79, 101] | <0.001*** |
| Comorbidities, n (%) |  |  |  |  |  |  |
| AMI, n (%) | 830 (19%) | 157 (14%) | 211 (19%) | 226 (20%) | 236 (21%) | <0.001*** |
| CABG, n (%) | 112 (2.5%) | 25 (2.3%) | 28 (2.5%) | 28 (2.5%) | 31 (2.8%) | 0.9 |
| CHF, n (%) | 698 (16%) | 166 (15%) | 179 (16%) | 195 (18%) | 158 (14%) | 0.15 |
| CKD, n (%) | 162 (3.7%) | 40 (3.6%) | 42 (3.8%) | 31 (2.8%) | 49 (4.4%) | 0.2 |
| COPD, n (%) | 462 (10%) | 90 (8.1%) | 122 (11%) | 124 (11%) | 126 (11%) | 0.035* |
| DM, n (%) | 1,398 (32%) | 168 (15%) | 255 (23%) | 390 (35%) | 585 (53%) | <0.001*** |
| OSAHS, n (%) | 335 (7.6%) | 51 (4.6%) | 75 (6.8%) | 85 (7.7%) | 124 (11%) | <0.001*** |
| PCI, n (%) | 356 (8.1%) | 68 (6.1%) | 73 (6.6%) | 101 (9.1%) | 114 (10%) | <0.001*** |
| Laboratory tests |  |  |  |  |  |  |
| BUN,mg/dL(median [IQR]) | 17 [13, 23] | 16 [12, 22] | 17 [13, 22] | 17 [13, 23] | 18 [14, 27] | <0.001*** |
| Chlorine,mmol/L,(median [IQR]) | 103.0 [100.0, 106.0] | 104.0 [101.0, 106.0] | 104.0 [101.0, 106.0] | 103.0 [100.0, 106.0] | 103.0 [99.0, 106.0] | <0.001*** |
| Potassium, mmol/L,(median [IQR]) | 4.00 [3.70, 4.40] | 4.00 [3.70, 4.30] | 4.00 [3.70, 4.30] | 4.00 [3.70, 4.40] | 4.10 [3.70, 4.50] | <0.001*** |
| Sodium,mmol/L,(median [IQR]) | 139.0 [137.0, 141.0] | 139.0 [137.0, 142.0] | 140.0 [138.0, 142.0] | 139.0 [137.0, 141.0] | 138.0 [136.0, 141.0] | <0.001*** |
| Glucose,mg/dL,(median [IQR]) | 122 [101, 159] | 100 [89, 115] | 114 [99, 135] | 132 [110, 163] | 174 [133, 238] | <0.001*** |
| Hb,g/dL,(median [IQR]) | 12.40 [10.90, 13.70] | 12.30 [11.00, 13.40] | 12.60 [11.10, 14.00] | 12.40 [10.80, 13.70] | 12.40 [10.78, 13.90] | 0.004** |
| HbA1c%, n (%) |  |  |  |  |  | <0.001*** |
| <5.7 | 887 (34%) | 332 (48%) | 282 (42%) | 189 (29%) | 84 (14%) |  |
| >6.4 | 726 (28%) | 65 (9.5%) | 102 (15%) | 204 (32%) | 355 (60%) |  |
| 5.7-6.4 | 980 (38%) | 288 (42%) | 284 (43%) | 251 (39%) | 157 (26%) |  |
| Missing | 1,825 | 422 | 434 | 461 | 508 |  |
| HDL,mg/dL, n (%) |  |  |  |  |  | <0.001*** |
| <45 | 1,691 (51%) | 278 (31%) | 402 (45%) | 492 (59%) | 519 (77%) |  |
| >55 | 846 (26%) | 398 (44%) | 247 (28%) | 142 (17%) | 59 (8.7%) |  |
| 45-55 | 759 (23%) | 222 (25%) | 241 (27%) | 199 (24%) | 97 (14%) |  |
| Missing | 1,122 | 209 | 212 | 272 | 429 |  |
| LDL,mg/dL, n (%) |  |  |  |  |  | <0.001*** |
| <=129 | 2,738 (85%) | 816 (91%) | 763 (86%) | 679 (82%) | 480 (80%) |  |
| >129 | 472 (15%) | 80 (8.9%) | 123 (14%) | 150 (18%) | 119 (20%) |  |
| Missing | 1,208 | 211 | 216 | 276 | 505 |  |
| TC,mg/dL,(median [IQR]) | 155 [132, 184] | 150 [126, 175] | 155 [133, 182] | 156 [133, 186] | 161 [137, 194] | <0.001*** |
| TG,mg/dL,(median [IQR]) | 116 [83, 171] | 69 [57, 83] | 103 [86, 121] | 139 [114, 168] | 242 [179, 335] | <0.001*** |
| Lymphocytes, %, n (%) |  |  |  |  |  | <0.001*** |
| <18 | 1,787 (70%) | 344 (62%) | 387 (65%) | 483 (72%) | 573 (77%) |  |
| >42 | 63 (2.5%) | 15 (2.7%) | 15 (2.5%) | 19 (2.8%) | 14 (1.9%) |  |
| 18-42 | 705 (28%) | 194 (35%) | 189 (32%) | 168 (25%) | 154 (21%) |  |
| Missing | 1,863 | 554 | 511 | 435 | 363 |  |
| Neutrophils, %, n (%) |  |  |  |  |  | <0.001*** |
| <34 | 49 (1.9%) | 7 (1.3%) | 12 (2.0%) | 19 (2.8%) | 11 (1.5%) |  |
| >71 | 1,755 (69%) | 352 (64%) | 386 (65%) | 467 (70%) | 550 (74%) |  |
| 34-71 | 751 (29%) | 194 (35%) | 193 (33%) | 184 (27%) | 180 (24%) |  |
| Missing | 1,863 | 554 | 511 | 435 | 363 |  |
| NT-probnp,pg/mL, n (%) |  |  |  |  |  | 0.6 |
| <=229 | 37 (11%) | 10 (14%) | 9 (14%) | 9 (9.0%) | 9 (9.4%) |  |
| >229 | 294 (89%) | 60 (86%) | 56 (86%) | 91 (91%) | 87 (91%) |  |
| Missing | 4,087 | 1,037 | 1,037 | 1,005 | 1,008 |  |
| PCO2, mmhg, (median [IQR]) |  |  |  |  |  | <0.001*** |
| <35 | 508 (23%) | 105 (27%) | 129 (27%) | 139 (24%) | 135 (19%) |  |
| >45 | 591 (27%) | 82 (21%) | 124 (26%) | 159 (27%) | 226 (31%) |  |
| 35-45 | 1,087 (50%) | 205 (52%) | 229 (48%) | 293 (50%) | 360 (50%) |  |
| Missing | 2,232 | 715 | 620 | 514 | 383 |  |
| PH (median [IQR]) |  |  |  |  |  | <0.001*** |
| <7.35 | 661 (29%) | 78 (19%) | 103 (21%) | 164 (27%) | 316 (43%) |  |
| >7.45 | 390 (17%) | 79 (19%) | 111 (22%) | 115 (19%) | 85 (11%) |  |
| 7.35-7.45 | 1,207 (53%) | 255 (62%) | 283 (57%) | 330 (54%) | 339 (46%) |  |
| Missing | 2,160 | 695 | 605 | 496 | 364 |  |
| PO2, mmhg, (median [IQR]) |  |  |  |  |  | 0.14 |
| <85 | 746 (34%) | 118 (30%) | 163 (34%) | 215 (36%) | 250 (35%) |  |
| >105 | 1,165 (53%) | 226 (58%) | 265 (55%) | 309 (52%) | 365 (51%) |  |
| 85-105 | 275 (13%) | 48 (12%) | 54 (11%) | 67 (11%) | 106 (15%) |  |
| Missing | 2,232 | 715 | 620 | 514 | 383 |  |
| PLT, K/uL, (median [IQR]) | 213 [169, 269] | 210 [166, 260] | 209 [169, 269] | 214 [171, 277] | 217 [167, 272] | 0.045* |
| WBC (median [IQR]) | 9.4 [7.1, 12.7] | 8.0 [6.4, 10.7] | 8.9 [6.9, 11.4] | 10.2 [7.5, 13.6] | 10.8 [7.9, 14.7] | <0.001*** |
| RBC (median [IQR]) | 4.12 [3.63, 4.55] | 4.05 [3.63, 4.42] | 4.17 [3.67, 4.60] | 4.14 [3.59, 4.58] | 4.17 [3.59, 4.65] | <0.001*** |
| PT, s, (median [IQR]) | 13.1 [11.9, 14.8] | 13.2 [12.0, 15.0] | 13.1 [11.9, 14.7] | 13.1 [12.0, 14.8] | 13.0 [11.9, 14.7] | 0.073 |
| Scr, mg/dL, (median [IQR]) | 0.90 [0.70, 1.10] | 0.90 [0.70, 1.10] | 0.90 [0.70, 1.10] | 0.90 [0.80, 1.10] | 1.00 [0.80, 1.30] | <0.001*** |
| TNT, μg/L, n (%) |  |  |  |  |  | 0.9 |
| <=0.01 | 96 (7.3%) | 21 (7.3%) | 19 (6.5%) | 28 (8.1%) | 28 (7.2%) |  |
| >0.01 | 1,225 (93%) | 268 (93%) | 275 (94%) | 319 (92%) | 363 (93%) |  |
| Missing | 3,097 | 818 | 808 | 758 | 713 |  |
| ACEI, n (%) | 1,836 (42%) | 425 (38%) | 461 (42%) | 479 (43%) | 471 (43%) | 0.085 |
| ARB, n (%) | 396 (9.0%) | 96 (8.7%) | 105 (9.5%) | 105 (9.5%) | 90 (8.2%) | 0.6 |
| Aspirin, n (%) | 2,714 (61%) | 699 (63%) | 672 (61%) | 702 (64%) | 641 (58%) | 0.032* |
| Betablocker, n (%) | 2,580 (58%) | 590 (53%) | 653 (59%) | 685 (62%) | 652 (59%) | <0.001*** |
| CCB, n (%) | 1,432 (32%) | 336 (30%) | 386 (35%) | 356 (32%) | 354 (32%) | 0.13 |
| Clopidogrel, n (%) | 872 (20%) | 204 (18%) | 209 (19%) | 237 (21%) | 222 (20%) | 0.3 |
| Statin, n (%) | 2,879 (65%) | 684 (62%) | 722 (66%) | 751 (68%) | 722 (65%) | 0.024* |
| Events |  |  |  |  |  |  |
| AKI3 | 2,513 (57%) | 518 (47%) | 600 (54%) | 670 (61%) | 725 (66%) | <0.001*** |
| RRT | 168 (3.8%) | 26 (2.3%) | 16 (1.5%) | 35 (3.2%) | 91 (8.2%) | <0.001*** |

Notes: ^1^ TyG index: Q1: 2.39–4.58; Q2: 4.58–4.80; Q3: 4.80–5.06; Q4: 5.06–7.28; ^2^ *P<0.05; **P<0.01; ***P<0.001; 3 AKI, as per the KDIGO guidelines, is described as an increase in SCr to at least 1.5 times the baseline within the last week, or an increase pf at least 0.3 mg/dl in SCr in 2 days, or urine output of less than 0.5 ml/kg/h for six or more hours.

# Supplementary Table 2. Baseline characteristics of the AKI group and other groups.

| **Variables** | **Overall** | **non-AKI** | **AKI** | **P-value^1^** |
| --- | --- | --- | --- | --- |
| Number of patients | 4,418 | 1,905 | 2,513 |  |
| Male (n%) | 2,501 (57%) | 1,042 (55%) | 1,459 (58%) | 0.026* |
| Age, years, (median [IQR]) | 67 [57, 78] | 68 [58, 78] | 67 [57, 78] | 0.2 |
| Race, n (%) |  |  |  | <0.001*** |
| Asian | 123 (2.8%) | 73 (3.8%) | 50 (2.0%) |  |
| Black | 407 (9.2%) | 228 (12%) | 179 (7.1%) |  |
| White | 2,799 (63%) | 1,254 (66%) | 1,545 (61%) |  |
| Other | 1,089 (25%) | 350 (18%) | 739 (29%) |  |
| BMI, kg/m2, n (%) |  |  |  | 0.8 |
| Normal  (18.5~23.9) | 447 (20%) | 239 (21%) | 208 (19%) |  |
| Obesity  (≥30) | 822 (37%) | 413 (36%) | 409 (38%) |  |
| Overweight  (23.9~29.9) | 879 (40%) | 452 (40%) | 427 (40%) |  |
| Underweight  (<18.5) | 56 (2.5%) | 29 (2.6%) | 27 (2.5%) |  |
| Missing | 2,214 | 772 | 1,442 |  |
| Disease severity score |  |  |  |  |
| APSIII (median [IQR]) | 36 [28, 47] | 33 [27, 39] | 40 [29, 54] | <0.001*** |
| SAPSII (median [IQR]) | 32 [26, 39] | 30 [25, 35] | 35 [27, 43] | <0.001*** |
| SOFA (median [IQR]) | 3.0 [1.9, 5.0] | 2.6 [1.6, 4.0] | 4.0 [2.0, 7.0] | <0.001*** |
| SIRS (median [IQR]) | 2.30 [2.00, 3.00] | 2.11 [1.92, 2.78] | 3.00 [2.00, 3.00] | <0.001*** |
| Vital Signs |  |  |  |  |
| SBP, mmhg, n (%) |  |  |  | 0.3 |
| <=140 | 1,821 (74%) | 893 (73%) | 928 (75%) |  |
| >140 | 640 (26%) | 329 (27%) | 311 (25%) |  |
| Missing | 1,957 | 683 | 1,274 |  |
| DBP, mmhg, n (%) |  |  |  | 0.2 |
| <=90 | 2,227 (90%) | 1,116 (91%) | 1,111 (90%) |  |
| >90 | 234 (9.5%) | 106 (8.7%) | 128 (10%) |  |
| Missing | 1,957 | 683 | 1,274 |  |
| HR (median [IQR]) | 84 [76, 95] | 83 [78, 90] | 86 [74, 100] | <0.001*** |
| Comorbidities, n (%) |  |  |  |  |
| AMI, n (%) | 830 (19%) | 313 (16%) | 517 (21%) | <0.001*** |
| CABG, n (%) | 112 (2.5%) | 27 (1.4%) | 85 (3.4%) | <0.001*** |
| CHF, n (%) | 698 (16%) | 262 (14%) | 436 (17%) | 0.001** |
| CKD, n (%) | 173 (3.9%) | 57 (3.0%) | 116(4.6%) | 0.006** |
| COPD, n (%) | 462 (10%) | 165 (8.7%) | 297 (12%) | <0.001*** |
| DM, n (%) | 1,425 (32.3%) | 571 (30%) | 854 (34%) | 0.006** |
| OSAHS, n (%) | 335 (7.6%) | 111 (5.8%) | 224 (8.9%) | <0.001*** |
| PCI, n (%) | 356 (8.1%) | 186 (9.8%) | 170 (6.8%) | <0.001*** |
| Laboratory tests |  |  |  |  |
| BUN, mg/dL (median [IQR]) | 17 [13, 23] | 17 [13, 22] | 18 [13, 25] | <0.001*** |
| Chlorine, mmol/L, (median [IQR]) | 103.0 [100.0, 106.0] | 103.0 [100.0, 106.0] | 103.0 [100.0, 106.0] | 0.6 |
| Potassium, mmol/L, (median [IQR]) | 4.00 [3.70, 4.40] | 4.00 [3.70, 4.30] | 4.10 [3.70, 4.40] | 0.027* |
| Sodium, mmol/L, (median [IQR]) | 139.0 [137.0, 141.0] | 139.0 [137.0, 141.0] | 139.0 [136.0, 141.0] | 0.056 |
| Glucose, mg/dL, (median [IQR]) | 122 [101, 159] | 114 [97, 147] | 129 [105, 168] | <0.001*** |
| Hb, g/dL, (median [IQR]) | 12.40 [10.90, 13.70] | 12.50 [11.10, 13.70] | 12.40 [10.80, 13.70] | 0.093 |
| HbA1c%, n (%) |  |  |  | 0.017* |
| <5.7 | 887 (34%) | 383 (33%) | 504 (36%) |  |
| >6.4 | 726 (28%) | 362 (31%) | 364 (26%) |  |
| 5.7-6.4 | 980 (38%) | 433 (37%) | 547 (39%) |  |
| Missing | 1,825 | 727 | 1,098 |  |
| HDL, mg/dL, n (%) |  |  |  | 0.005** |
| <45 | 1,691 (51%) | 777 (48%) | 914 (54%) |  |
| >55 | 846 (26%) | 436 (27%) | 410 (24%) |  |
| 45-55 | 759 (23%) | 392 (24%) | 367 (22%) |  |
| Missing | 1,122 | 300 | 822 |  |
| LDL, mg/dL, n (%) |  |  |  | 0.7 |
| <=129 | 2,738 (85%) | 1,330 (85%) | 1,408 (86%) |  |
| >129 | 472 (15%) | 234 (15%) | 238 (14%) |  |
| Missing | 1,208 | 341 | 867 |  |
| TC, mg/dL, (median [IQR]) | 155 [132, 184] | 159 [137, 189] | 152 [130, 178] | <0.001*** |
| TG, mg/dL, (median [IQR]) | 116 [83, 171] | 110 [78, 160] | 121 [85, 183] | <0.001*** |
| Lymphocytes, %, n (%) |  |  |  | <0.001*** |
| <18 | 1,787 (70%) | 479 (57%) | 1,308 (76%) |  |
| >42 | 63 (2.5%) | 34 (4.0%) | 29 (1.7%) |  |
| 18-42 | 705 (28%) | 328 (39%) | 377 (22%) |  |
| Missing | 1,863 | 1,064 | 799 |  |
| Neutrophils, %, n (%) |  |  |  | <0.001*** |
| <34 | 49 (1.9%) | 20 (2.4%) | 29 (1.7%) |  |
| >71 | 1,755 (69%) | 497 (59%) | 1,258 (73%) |  |
| 34-71 | 751 (29%) | 324 (39%) | 427 (25%) |  |
| Missing | 1,863 | 1,064 | 799 |  |
| NT-probnp, pg/mL, n (%) |  |  |  | 0.3 |
| <=229 | 37 (11%) | 12 (14%) | 25 (10%) |  |
| >229 | 294 (89%) | 74 (86%) | 220 (90%) |  |
| Missing | 4,087 | 1,819 | 2,268 |  |
| PCO2, mmhg, (median [IQR]) |  |  |  | 0.6 |
| <35 | 508 (23%) | 83 (22%) | 425 (24%) |  |
| >45 | 591 (27%) | 98 (26%) | 493 (27%) |  |
| 35-45 | 1,087 (50%) | 197 (52%) | 890 (49%) |  |
| Missing | 2,232 | 1,527 | 705 |  |
| PH (median [IQR]) |  |  |  | 0.009** |
| <7.35 | 661 (29%) | 96 (23%) | 565 (31%) |  |
| >7.45 | 390 (17%) | 74 (18%) | 316 (17%) |  |
| 7.35-7.45 | 1,207 (53%) | 244 (59%) | 963 (52%) |  |
| Missing | 2,160 | 1,491 | 669 |  |
| PO2, mmhg, (median [IQR]) |  |  |  | 0.2 |
| <85 | 746 (34%) | 132 (35%) | 614 (34%) |  |
| >105 | 1,165 (53%) | 209 (55%) | 956 (53%) |  |
| 85-105 | 275 (13%) | 37 (9.8%) | 238 (13%) |  |
| Missing | 2,232 | 1,527 | 705 |  |
| PLT, K/uL, (median [IQR]) | 213 [169, 269] | 219 [176, 276] | 207 [161, 263] | <0.001*** |
| WBC (median [IQR]) | 9.4 [7.1, 12.7] | 8.3 [6.5, 10.9] | 10.4 [7.7, 14.1] | <0.001*** |
| RBC (median [IQR]) | 4.12 [3.63, 4.55] | 4.16 [3.69, 4.55] | 4.09 [3.57, 4.56] | 0.003** |
| PT, s, (median [IQR]) | 13.1 [11.9, 14.8] | 13.0 [11.9, 14.4] | 13.2 [11.9, 15.1] | <0.001*** |
| Scr, mg/dL, (median [IQR]) | 0.90 [0.70, 1.10] | 0.90 [0.70, 1.10] | 0.90 [0.80, 1.20] | <0.001*** |
| TNT, μg/L, n (%) |  |  |  | 0.14 |
| <=0.01 | 96 (7.3%) | 38 (8.8%) | 58 (6.5%) |  |
| >0.01 | 1,225 (93%) | 394 (91%) | 831 (93%) |  |
| Missing | 3,097 | 1,473 | 1,624 |  |
| ACEI, n (%) | 1,836 (42%) | 833 (44%) | 1,003 (40%) | 0.011* |
| ARB, n (%) | 396 (9.0%) | 181 (9.5%) | 215 (8.6%) | 0.3 |
| Aspirin, n (%) | 2,714 (61%) | 1,261 (66%) | 1,453 (58%) | <0.001*** |
| Betablocker, n (%) | 2,580 (58%) | 988 (52%) | 1,592 (63%) | <0.001*** |
| CCB, n (%) | 1,432 (32%) | 494 (26%) | 938 (37%) | <0.001*** |
| Clopidogrel, n (%) | 872 (20%) | 464 (24%) | 408 (16%) | <0.001*** |
| Statin, n (%) | 2,879 (65%) | 1,294 (68%) | 1,585 (63%) | <0.001*** |
| Event |  |  |  |  |
| RRT | 168 (3.8%) | 1 (<0.1%) | 167 (6.6%) | <0.001*** |
| TyG | 4.80 [4.58, 5.06] | 4.73 [4.53, 4.98] | 4.84 [4.63, 5.12] | <0.001*** |

Notes: ^1^*P<0.05; **P<0.01; ***P<0.001.
